# Blue Light (λ = 453 nm) Significantly Reduces TGF-β-Induced Fibroblast Differentiation Through Reversible Disruption of Mitochondrial Respiration, Glycolysis, and ATP Production Rate

**DOI:** 10.3390/biomedicines13092231

**Published:** 2025-09-10

**Authors:** Pia Steentjes, Julia Krassovka, Christoph V. Suschek, Uwe Maus, Lisa Oezel

**Affiliations:** Department for Orthopedics and Trauma Surgery, Medical Faculty and University Hospital Düsseldorf, Heinrich Heine University Düsseldorf, Moorenstr. 5, 40225 Düsseldorf, Germanylisa.oezel@med.uni-duesseldorf.de (L.O.)

**Keywords:** myofibroblast differentiation, blue light (453 nm), alpha-smooth muscle actin (αSMA), glycolysis, mitochondrial respiration, ATP, cellular energy metabolism

## Abstract

**Background/Objectives**: Abnormal differentiation of human skin fibroblasts into myofibroblasts contributes to fibrotic skin disorders such as hypertrophic scars, keloids, and Dupuytren’s disease. This process is characterized by increased fibroblast proliferation, enhanced differentiation into myofibroblasts, and reduced programmed cell death (apoptosis). We previously demonstrated that blue light irradiation (λ = 453 nm) significantly and dose-dependently inhibits both spontaneous and TGF-β-induced fibroblast differentiation. **Methods**: Because fibroblast differentiation depends on cellular energy metabolism, we investigated whether the inhibitory effect of blue light is linked to changes in the cells’ energy balance. **Results**: We found that blue light reduced TGF-β-induced differentiation, as shown by decreased levels of α-SMA and EDA-fibronectin, key markers of myofibroblast formation. This effect was strongly associated with almost complete inhibition of mitochondrial respiration, reduced glycolysis, a lower NAD^+^/NADH ratio, and decreased ATP production. ATP-dependent processes, including endocytosis and lysosomal activity, both essential parameters of fibroblast differentiation, were also strongly suppressed. Importantly, all these changes were fully reversible within 24 h after the last irradiation. **Conclusions**: Mechanistically, we propose that blue light triggers photochemical reduction in flavins in proteins of the respiratory chain and possibly the Krebs cycle, which temporarily alters cellular energy metabolism. These findings suggest that non-toxic blue light therapy (80 J/cm^2^) can effectively prevent factor-induced fibroblast differentiation and may serve as a standalone or supportive treatment to reduce fibrotic events such as scarring and keloid formation. Furthermore, our results indicate that targeting cellular energy metabolism, whether physically or pharmacologically, could be a promising strategy to prevent sclerotic skin disorders.

## 1. Introduction

Fibroproliferative and sclerotic diseases, such as Dupuytren’s disease and excessive scar tissue formation, are significant health concerns. Excessive scar formation is often linked to disordered tissue remodeling, primarily involving fibrogenesis and myofibroblast differentiation. Despite differences in etiology and clinical presentation, fibrotic disorders share a common basis: overproduction of growth factors, proteolytic enzymes, angiogenic/fibrogenic factors, and cytokines [1,2]. These factors drive fibroblast-to-myofibroblast differentiation, leading to tissue remodeling and fibrosis.

Myofibroblasts originate from mesenchymal cells, epithelial cells, or bone marrow-derived mesenchymal stem cells [3]. They exhibit increased contractility due to stress fibers such as α-smooth muscle actin (α-SMA) [2,4]. Once activated, myofibroblasts mediate connective tissue repair and remodeling. However, excessive activity leads to pathological fibrosis and scar tissue formation. Key features include increased fibroblast migration, proliferation, and TGF-β-induced differentiation, along with heightened collagen production and reduced apoptosis rates. TGF-β, a critical growth factor, stimulates fibroblast proliferation and myofibroblast differentiation [5].

Therapies for sclerotic skin disorders include X-ray, visible light [6,7,8], cortisone, immunosuppressants [9], laser therapy, cryotherapy [10], and surgical removal [11]. High recurrence rates underscore the need for effective therapies and preventive measures. Silicone-based products (gels and sheets) show efficacy in scar prevention, particularly for individuals prone to keloids, due to their ease of use and proven benefits [12]. Advances in wound healing research have identified inflammation, epithelial–mesenchymal transition, and myofibroblast activity as key targets for fibrosis prevention [13]. Corticosteroids remain the standard treatment for keloids and hypertrophic scars, though their broader role in scar prevention requires further study [14]. Emerging molecular mediators, such as IL-4/IL-13 axis inhibitors and TGF-β pathway modulators, offer promising antifibrotic strategies [15]. Mechanotherapy, which involves pressure therapies and tension-offloading techniques, also shows potential for scar optimization [16].

Enhanced aerobic glycolysis plays a pivotal role in myofibroblast differentiation and contractile functions driven by TGF-β [17,18,19]. This metabolic reprogramming supports cellular growth and differentiation by regulating biosynthetic pathways critical for fibroblast function [20]. Glycolytic remodeling was first observed in cystic fibrosis patients, where increased glycolysis and glucose metabolism enzyme activity were evident [20,21,22]. Subsequent studies confirmed similar shifts in other fibroblast populations [23,24]. Transcriptional upregulation of glycolytic genes, many of which serve as metabolic checkpoints, promotes this glycolytic shift [23,25,26]. Targeting glycolysis could thus offer a therapeutic strategy for fibrotic diseases [18,27]. For example, glucose metabolism inhibitors like 2-deoxyglucose (2DG) effectively prevent α-SMA expression [23,26], reducing ATP production, arresting cell cycles, and inhibiting cell growth [28,29]. However, other measures that ultimately led to a significant reduction in fibroblast differentiation, as we recently demonstrated in a study involving CO_2_-containing solutions, correlated with significantly reduced glycolysis, as well as decreased mitochondrial respiration and ATP synthesis rates [30].

Blue light at 453 nm has a strong photo-reductive property toward flavins, which play an essential role in pivotal energy-producing systems of the cell and serve as photo-acceptors in this spectral range. Blue light might therefore be capable of modulating cellular energy metabolism through this mechanism in fibroblasts. Indeed, from previous studies, we know that blue light can reduce oxidative respiration, glycolysis, and consequently the ATP production rate in several tumor cells [31,32]. From other studies of our own, we know that blue light can also significantly reduce fibroblast differentiation in human skin fibroblasts [8,33,34]. Therefore, it seems plausible to establish a causality between these two events in fibroblasts as well, which could be of great relevance for the development of new therapeutic options in the prophylaxis of fibrosis-related events in human skin.

## 2. Materials and Methods

### 2.1. Materials

Unless otherwise stated, all chemicals, antibodies, and assay kits were sourced from Sigma-Aldrich Chemie GmbH (Munich, Germany).

### 2.2. Cell Culture

#### 2.2.1. Skin Sample Collection

Primary human dermal fibroblasts were isolated from skin specimens obtained with informed consent from twelve healthy donors (eight female, four male; age range: 27–66 years) undergoing elective breast reduction or abdominoplasty surgery. For all experiments, fibroblasts from each donor were cultured and analyzed independently. At no point were cells pooled across donors.

#### 2.2.2. Fibroblast Isolation

The cells were cultured under standardized conditions as previously described [8]. Briefly, skin samples were placed in a Petri dish, cut into small pieces, and incubated overnight at 4 °C in Dispase II solution. The following day, the epidermis and dermis were separated, and the dermis containing fibroblasts was treated with collagenase for 60 min at 37 °C in a temperature-controlled shaker. The digested dermis was then vortexed, passed through a mesh (50–100 µM), and fibroblasts were isolated. After centrifugation, the supernatant was discarded, and the cell pellet was resuspended in 10 mL of fresh DMEM. The isolated fibroblasts were transferred into culture flasks and incubated at 37 °C with 5% CO_2_ to allow growth.

#### 2.2.3. Ethics Approval

The study protocol, including the use of human tissue, was approved by the Ethics Committee of the Medical Faculty at Heinrich-Heine-University Düsseldorf (study number: 3634) and adhered to the principles of the Declaration of Helsinki.

### 2.3. Irradiation of Human Skin Fibroblast Cultures with Blue Light

#### 2.3.1. LED Device and Setup

LED arrays for this study were supplied by Philips Research (Aachen, Germany). A narrow-band LED device (12 × 10 cm array, 60 LEDs) was used, with irradiance characterized using an integrating sphere. Electrical and optical parameters were as follows: current = 1.75 A, voltage = 40 VDC, input power = 69 W, optical output power = 13 W, LED max intensity = 0.21 W/nm at 453 nm, and emission bandwidth ±7 nm.

#### 2.3.2. Irradiation Protocol

During irradiation, the LED array was positioned 5 cm above the monolayer, delivering 39 mW/cm^2^. Exposure duration was 2050 s for a total fluence of 80 J/cm^2^. Sample temperature remained below 33 °C. Control plates were placed in a 33 °C heating cabinet for 45 min without irradiation. Additional controls confirmed negligible osmotic effects.

### 2.4. Immunocytochemistry

#### 2.4.1. Cell Fixation and Permeabilization

Cells were fixed with 4% paraformaldehyde for 15 min. Following permeabilization with 0.2% Triton X-100, FBs were treated with PBS/BSA (4%) blocking buffer (PAA, Colbe, Germany) for 30 min.

#### 2.4.2. Primary Antibody Incubation

Subsequently, the cells were incubated for 60 min with a monoclonal mouse anti-human α-smooth muscle actin (αSMA, ab7817, Abcam, Cambridge, UK) antibody diluted at 1:400 in the blocking buffer. After three washing steps, αSMA staining was visualized using the Dako kit (DakoCytomation, Hamburg, Germany).

#### 2.4.3. Image Acquisition and Quantification

Photographs of each well were captured to assess myofibroblast differentiation [34]. Quantitative analysis of α-SMA-positive myofibroblasts was carried out using Western blotting.

### 2.5. Western Blotting

#### 2.5.1. Protein Collection and Lysate Preparation

Fibroblast cultures were rinsed with ice-cold PBS (4 °C), and cell lysates were obtained by adding 40 µL of RIPA lysis buffer supplemented with 14.2% protease inhibitor (7×; Roche, Basel, Switzerland). The lysates were further sonicated, and the supernatants were collected following centrifugation at 20,000× *g* for 10 min.

#### 2.5.2. Electrophoresis and Transfer

A total of 30 µg of protein per sample was loaded onto SDS-PAGE gels, followed by transfer to nitrocellulose membranes (Peqlab, Erlangen, Germany). Membranes were blocked overnight (16 h) at 4 °C in T-TBS containing 5% non-fat milk and 0.1% Tween 20. Primary antibodies were then applied according to the respective manufacturer’s instructions.

#### 2.5.3. Antibody Detection and Analysis

For Western blot analysis of protein expression, the following rabbit anti-human antibodies were used as primary antibodies: EDA-FN (ab6328, Abcam, Cambridge, UK), GAPDH (IMG-6665A, Novus Biologicals, Cambridge, UK), α-tubulin (#sc-8035, Santa Cruz Biotechnology, Dallas, TX, USA), and α-smooth muscle actin (ab7817, Abcam, Cambridge, UK). Western blotting was carried out using the XcellSureLock Mini-Cell System (Invitrogen, Karlsruhe, Germany) under reducing conditions. Protein concentrations in the samples were measured prior to blotting using the Pierce™ BCA Protein Assay Kit (#23225, Thermo Fisher, Dreieich, Germany).

After three washing steps (5 min each in T-TBS), membranes were incubated for one hour with secondary antibodies diluted at 1:1000: either goat anti-mouse IgG/HRP (#P0447) or goat anti-rabbit IgG/HRP (#P0449) from Agilent Technologies (Santa Clara, CA, USA). The Clarity™ Western ECL Substrate (BioRad, Hercules, CA, USA, #170-5060) was used for detection after an additional four washing steps (3 min each in T-TBS). Signal intensities were evaluated using the Quantity One 1-D Analysis Software, version 4.6.5 (BioRad).

### 2.6. Photoreduction of Flavin Adenine Dinucleotide

Flavin adenine dinucleotide (FAD), functioning as a cofactor and redox agent, is essential for cellular metabolism and enzymatic activities. The redox forms of FAD, FADH, and FADH_2_ exhibit distinct absorbance spectra. To investigate potential photoreduction, FAD solutions (10 mM, BioVision, Milpitas, CA, USA) prepared in distilled water were exposed to blue light (0–100 J/cm^2^). The absorbance spectra were then recorded in the range of 300–500 nm using a photometer (Specord 205, Analytik Jena, Jena, Germany).

### 2.7. Detection of NAD^+^/NADH

To quantify intracellular NAD^+^ and NADH levels and calculate their ratio, we used the NAD^+^/NADH detection kit from Abcam (ab65348, Cambridge, UK) and conducted the experiment exactly according to the manufacturer’s instructions.

### 2.8. Seahorse^®^ Assay

#### 2.8.1. OCR Measurement

To determine the oxygen consumption rate (OCR) and investigate various parameters of mitochondrial respiration, measurements were performed using the Agilent Seahorse XF24 Extracellular Flux Analyzer (Seahorse Bioscience, North Billerica, MA, USA) [35], utilizing the Mito Stress Test Assay (Seahorse Bioscience, North Billerica, MA, USA) according to the protocol described by Butler et al. [36].

#### 2.8.2. ECAR Measurement

Additionally, the same device was used to examine the extracellular acidification rate (ECAR) of cell cultures with the Extracellular Acidification Rate (ECAR) Assay from the company, which serves as an indicator of the energetic metabolic pathways of glycolysis [37].

#### 2.8.3. Data Analysis

The analysis of OCR and ECAR in untreated as well as light-exposed tumor cell cultures was conducted under the same conditions as previously described in an earlier study by us [31].

### 2.9. Cellular ATP Content

To further investigate the mechanism of cell death induced by chemotherapy, with the additional influence of blue light irradiation, we measured ATP levels in the treated cells. For this, we utilized the ATP assay (ATP Kit #LBR-T010, Biaffin GmbH & Co KG; Kassel, Germany), following the guidelines provided by the manufacturer.

### 2.10. Endocytosis and Lysosomal Activity—Neutral-Red Assay

Endocytosis and lysosomal activity were determined using the Neutral-Red assay [38]. The respective FB cultures were incubated for 90 min with Neutral Red (1:100 dilution of a 3% solution), washed twice with PBS, then microscopic photographs were taken, the wells were dried completely, and lysed with isopropanol containing 0.5% of 1N HCl. Extinction of the supernatants, which represent a function of endocytosis and lysosomal activity, was then measured at 530 nm.

### 2.11. Statistical Analysis

Statistical analyses were performed using GraphPad Prism 8 (San Diego, CA, USA). To assess significant differences, we applied one-way ANOVA followed by post hoc multiple comparison tests (Tukey method) or, alternatively, the Wilcoxon test or Student’s *t*-test. A *p*-value of less than 0.05 was regarded as statistically significant.

## 3. Results

### 3.1. Impact of Blue Light (453 nm) on Fibroblast Viability and Differentiation

**Figure 1:**
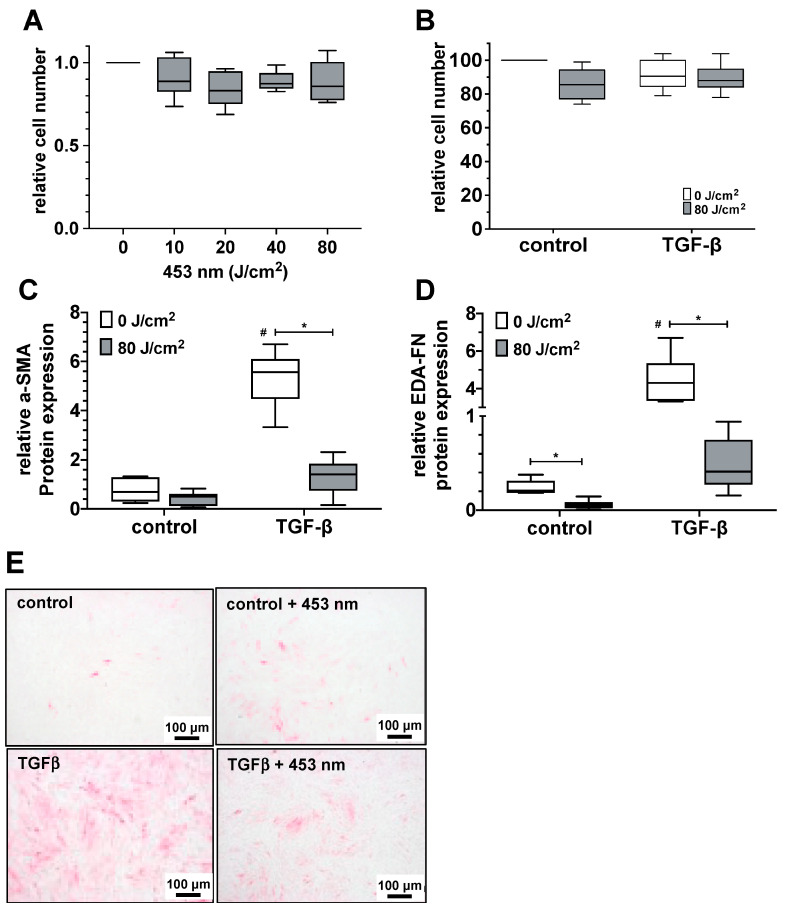
Impact of blue light (453 nm) on fibroblast viability and differentiation. (**A**) Cell viability (relative cell number) of primary human skin fibroblast cultures 24 h after exposure to blue light (453 nm) at doses indicated. The values of 8 individual (n = 8) experiments are shown as boxplots with median values and with whiskers with minimum and maximum. (**B**) Cell viability (relative cell number) of TGF-β activated and myofibroblasts differentiated primary human skin fibroblast cultures 24 h after exposure to blue light (453 nm) at the indicated dose. The values of 6 individual (n = 6) experiments are shown as boxplots with median values and with whiskers with minimum and maximum. (**C**,**D**) Effect of blue light (80 J/cm^2^, 453 nm, gray boxplots) on myofibroblast differentiation of resting (control, white boxplots) or TGF-β-activated primary human skin fibroblast cultures was evaluated by protein quantification of the differentiation markers α-SMA (**C**) and EDA-FN (**D**). The relative protein expression was quantified by Western blot 24 h after the last irradiation. In (**C**), the values of 8 individual experiments (*n* = 8) and in (**D**) of 6 individual experiments (*n* = 6) are shown as boxplots with median values and whiskers representing minimum and maximum. ^#^, *p* < 0.05 as compared to the untreated control cultures. *, *p* < 0.05 as compared to the respective non-irradiated TGF-β-activated cultures. (**E**) Exemplary photographs of an individual experiment for the immunocytochemical visualization of α-SMA expression (red signal) in non-irradiated or light-exposed (80 J/cm^2^, 453 nm) control and TGF-β-activated fibroblast cultures detected 24 h after the last irradiation.

As parameters of myofibroblast differentiation, we quantified the protein expression of differentiation markers α-smooth muscle actin (α-SMA) and extra domain A-fibronectin (EDA-FN). As shown in Figure 1A,B, irradiation with blue light (453 nm) at light doses up to 80 J/cm^2^ did not lead to statistically significant changes in cell viability of primary human skin fibroblast cultures (Figure 1A), nor of myofibroblast cultures after TGF-β activation of fibroblasts (Figure 1B). However, exposure of fibroblast cultures to blue light at 80 J/cm^2^ resulted in a strong and statistically significant reduction in α-SMA expression in TGF-β-activated cells (Figure 1C,E), as well as EDA-FN expression in resting and TGF-β-activated fibroblast cultures (Figure 1D).

### 3.2. Photoreduction of FAD and Reduced NAH^+^/NADH-Ratio in Light (453 nm) Exposed Fibroblast Cultures

**Figure 2:**
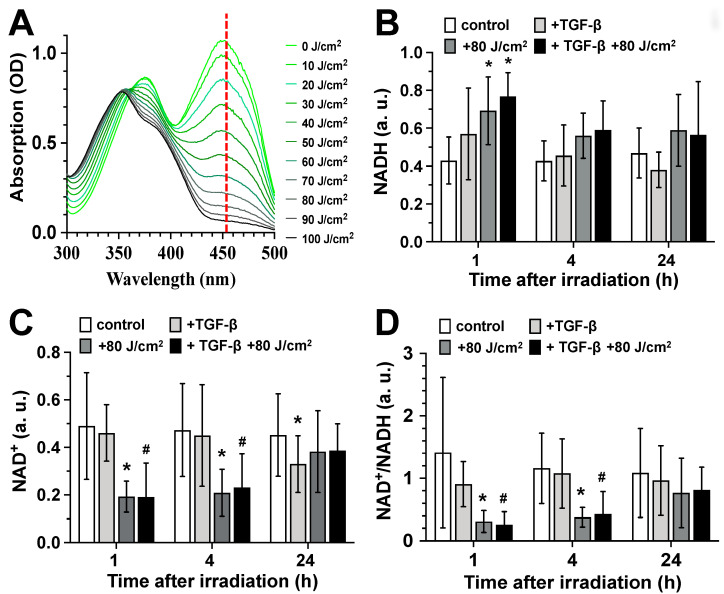
Photoreduction of FAD and modulation of the NAH^+^/NADH-ratio in light (453 nm) exposed fibroblast cultures. (**A**) Absorption spectra (300–500 nm) of flavin adenine dinucleotide solutions (10 mM) after irradiation with blue light (453 nm; 10–100 J/cm^2^). The top spectrum line with the highest OD value at 450 nm (0 J/cm^2^) represents the oxidized form of FAD. The OD value of the absorption peak at 450 nm decreases with the proportion of photo-reduced FAD form. (**B**–**D**) Relative values of NADH (**B**), NAD^+^ (**C**), and NAD^+^/NADH-ratio (**D**) in control fibroblast cultures (white bars), TGF-β-activated cultures (light gray), irradiated control cultures (dark gray), and irradiated TGF-β-activated cultures (black bars) were detected 1, 4, and 24 h after the light exposure (453 nm, 80 J/cm^2^). Bars represent the mean ± S.D. of 6 individual experiments (n = 6). *, *p* < 0.05 as compared to non-irradiated control cultures (white bars); ^#^, *p* < 0.05 as compared to non-irradiated TGF-β-activated cultures (light gray).

Irradiation of a flavin adenine dinucleotide (FAD, 10 mM) containing solution with blue light (453 nm; 0–100 J/cm^2^), we observed a strong and light dose-dependent increase in the appearance of photo-reduced FAD, here seen in the form of a light dose-dependent decrease in the absorption at 450 nm (Figure 2A).

Additionally, resting as well as TGF-β-activated human skin fibroblasts irradiation with 80 J/cm^2^ of blue light led to a statistically significant increase in intracellular NADH amount (Figure 2B), a decreased NAD^+^ concentration (Figure 2C), and thus to a strongly reduced NAD^+^/NADH-ratio (Figure 2D) at the time point 1 h after irradiation. At the time point of 4 h post-irradiation, the NADH levels in the irradiated cultures were still elevated, though not significantly, while the NAD^+^ levels remained significantly decreased, as did the NAD^+^/NADH ratio (Figure 2B–D). By 24 h post-irradiation, the concentrations of NAD^+^ and NADH returned to the levels observed in the unirradiated controls (Figure 2B–D).

### 3.3. Modulation of Mitochondrial Respiration, Glycolysis, and ATP Metabolism by Blue Light (453 nm)

**Figure 3:**
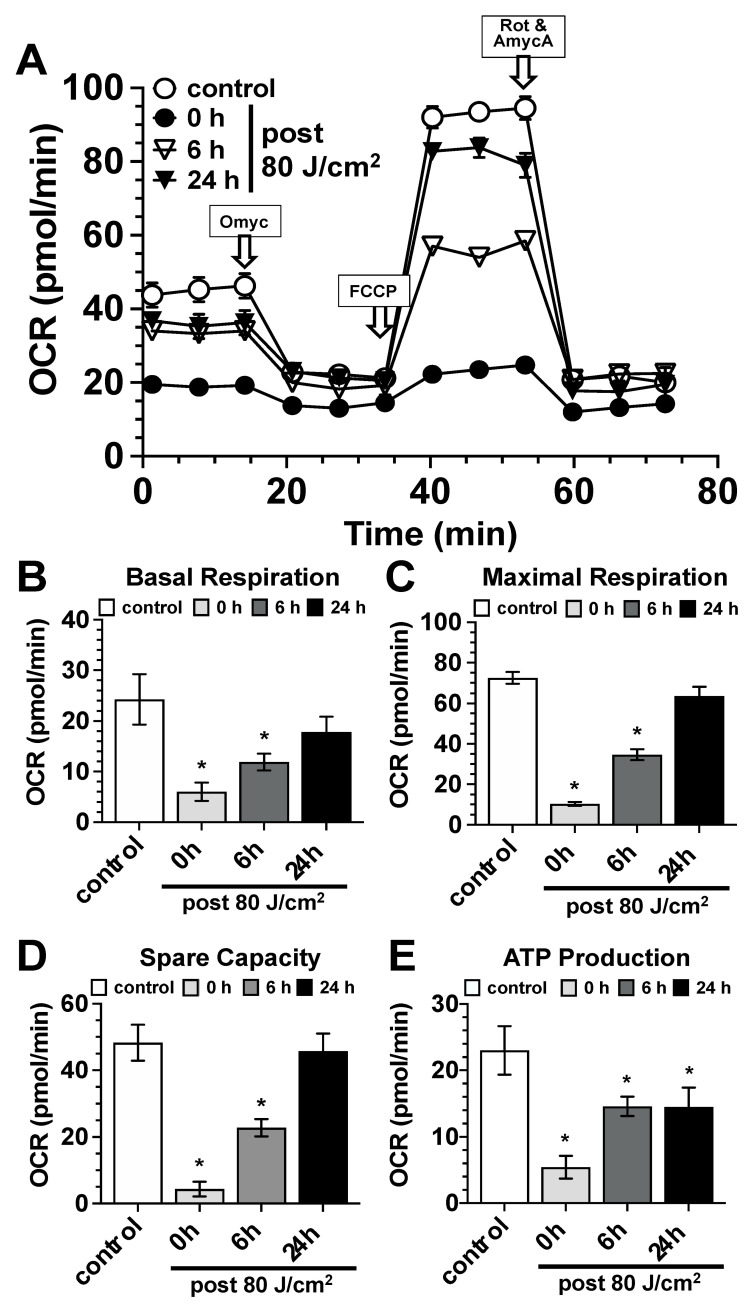
Modulation of mitochondrial respiration by blue light (453 nm). (**A**) Seahorse^®^ analysis (Seahorse XF Cell Mito Stress Test) of the impact of blue light exposure (453 nm, 80 J/cm^2^) on parameters of mitochondrial respiration (OCR–Oxygen Consumption Rate in pmol/min) of control fibroblast cultures (white circles) and irradiated fibroblast cultures immediately after (0 h, black circles), 6 h (white triangles), and 24 h after exposure (black triangles). Arrows indicate the time points of the addition of the assay-specific inhibitors, oligomycin (Omyc), carbonyl cyanide-*p*-trifluoromethoxyphenylhydrazone (FCCP), and rotenone and antimycin A (Rot and AmycA). Values represent the mean ± S.D. of six individual experiments. The values shown in (**A**) were used to calculate the parameters for basal respiration (**B**), maximal respiration (**C**), spare capacity (**D**), and ATP production (**E**) of control fibroblast cultures (white bars) and irradiated fibroblast cultures immediately after (0 h, light gray bars), 6 h (dark gray bars), and 24 h after exposure (black bars). Bars represent the mean ± S.D. of 6 individual experiments (n = 6). *, *p* < 0.05 as compared to the non-treated control cultures.

**Figure 4:**
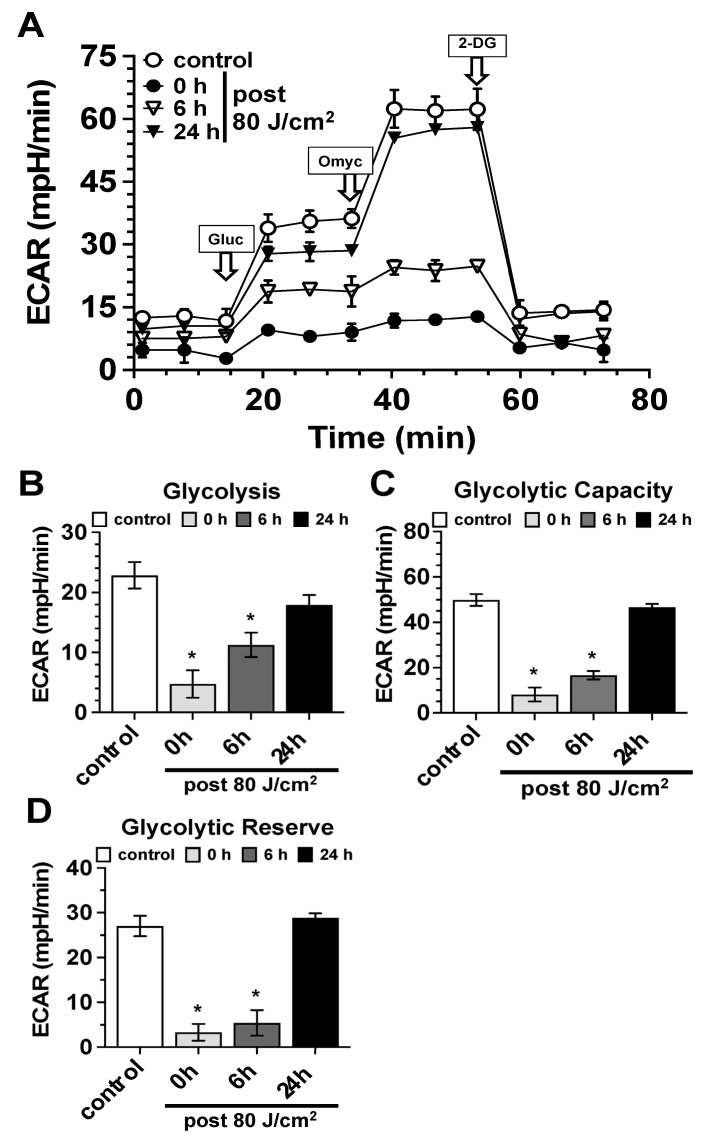
Modulation of glycolysis by blue light (453 nm). (**A**) Seahorse^®^ analysis (Seahorse XF Glycolysis Stress Test) of the impact of blue light exposure (453 nm, 80 J/cm^2^) on parameters of glycolysis (ECAR–Extracellular Acidification Rate in mpH/min) of control fibroblast cultures (white circles) and irradiated fibroblast cultures immediately after (0 h, black circles), 6 h (white triangles), and 24 h after exposure (black triangles). Arrows indicate the time points of the addition of the assay-specific inhibitors D-glucose (Gluc), oligomycin (Omyc), and 2-deoxy-glucose (2-DG). Values represent the mean ± S.D. of six individual experiments. The values shown in A were used to calculate the parameters for glycolysis (**B**), glycolytic capacity (**C**), and glycolytic reserve (**D**) of control fibroblast cultures (white bars) and irradiated fibroblast cultures immediately after (0 h, light gray bars), 6 h (dark gray bars), and 24 h after exposure (black bars). Bars represent the mean ± S.D. of 6 individual experiments (n = 6). *, *p* < 0.05 as compared to the non-treated control cultures.

**Figure 5:**
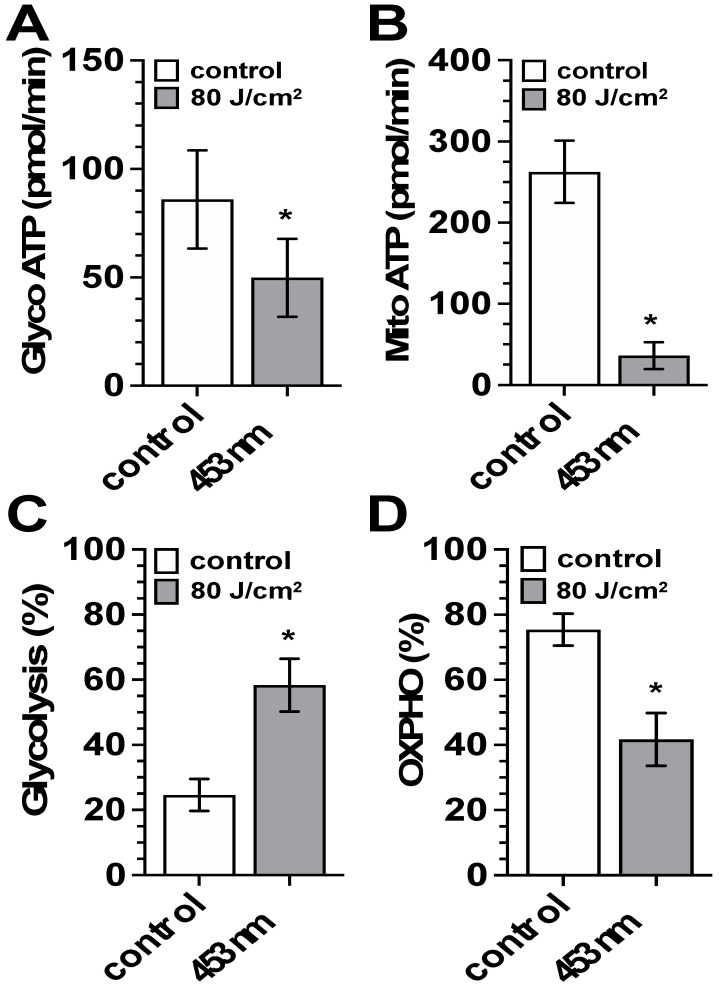
Modulation of ATP metabolism by blue light (453 nm). (**A**,**B**) Seahorse^®^ analysis (Seahorse XF Real-Time ATP Rate Assay) of the impact of blue light exposure (453 nm, 80 J/cm^2^) on (**A**) glycolytic ATP production rate (pmol/min) and (**B**) mitochondrial ATP production rate (pmol/min) of control fibroblast cultures (white bars) and irradiated fibroblast cultures (453 nm) one hour after light exposure (gray bars). (**C**,**D**) The percentage contribution of glycolytic (**C**) and mitochondrial (**D**) ATP production to the total ATP production rate of control fibroblast cultures (white bars) and irradiated fibroblast cultures (453 nm) one hour after light exposure (gray bars). Bars represent the mean ± S.D. of six individual experiments. *, *p* < 0.05 as compared to the non-treated control cultures.

Mitochondrial respiration: Using Agilent’s Seahorse^®^ technology, we evaluated the impact of blue light exposure on parameters of mitochondrial respiration (Figure 3), glycolysis (Figure 4), and ATP metabolism (Figure 5). Regarding mitochondrial respiration, irradiation with 80 J/cm2 led to a significant and statistically significant decrease in parameters for basal respiration (Figure 3A,B), maximal respiration (Figure 3A,C), spare capacity (Figure 3A,D), and ATP production (Figure 3A,E). These reductions were observed immediately after irradiation and persisted up to 6 h post-irradiation, with values mostly returning to baseline levels 24 h after light exposure. An exception was the ATP value parameter, which was still slightly, though statistically significantly, reduced 24 h after irradiation (Figure 3E).

Glycolysis: As part of the characterization of the modulation of glycolysis by blue light, we observed, similar to the effects described above on mitochondrial respiration, a clear impact of light exposure on glycolysis (Figure 4). Irradiation of human skin fibroblast cultures with 80 J/cm^2^ induced a strong and statistically significant reduction in glycolysis (Figure 4A,B), glycolytic capacity (Figure 4A,C), and glycolytic reserve (Figure 4A,D). This significant reduction was most pronounced immediately after light exposure and lasted for at least 6 h post-irradiation. As with glycolysis, the described inhibitory effects were fully reversible, with all mentioned parameters returning to the values of the unirradiated controls 24 h after light treatment (Figure 4).

ATP metabolism: An immediate effect of blue light-induced reduction in the parameters of the mitochondrial respiratory chain and glycolysis was a strong and statistically significant decrease in the Seahorse^®^ Values for glycolytic ATP production rate by approximately 50% (Figure 5A) and the mitochondrial ATP production rate by more than 80% (Figure 5B). Regardless of the reduced light-induced ATP production rates, exposure to blue light caused a percentage increase in the share of glycolytic ATP production (Figure 5C) and a significant decrease in the percentage share of mitochondrial ATP production (Figure 5D).

### 3.4. Reduction in Cellular ATP Production and ATP-Dependent Lysosomal Activity by Exposure to Blue Light (453 nm)

**Figure 6:**
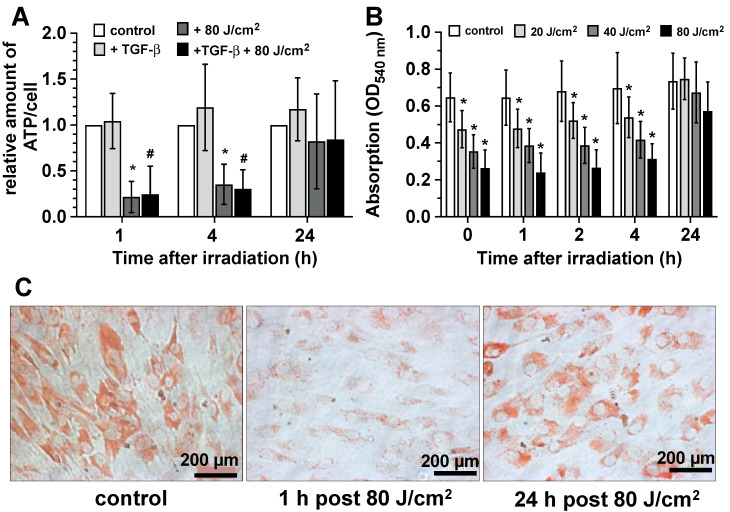
Modulation of ATP metabolism by blue light (453 nm). (**A**) Impact of blue light exposure on cellular ATP production in control fibroblast cultures (white bars), TGF-β-activated cultures (light gray bars), irradiated control cultures (dark gray bars), and irradiated TGF-β-activated cultures (black bars) detected 1, 4, and 24 h after the light exposure (453 nm, 80 J/cm^2^). Bars represent the mean ± S.D. of 8 individual experiments (n = 8). *, *p* < 0.05 as compared to non-irradiated control cultures (white bars); ^#^, *p* < 0.05 as compared to non-irradiated TGF-β-activated cultures (light gray). (**B**) Impact of blue light exposure on ATP-dependent endocytosis of neutral red and lysosomal activity of control fibroblast cultures (white bars) and fibroblast cultures irradiated with 20 J/cm^2^ (light gray bars), 40 J/cm^2^ (dark gray bars), and 80 J/cm^2^ (black bars) detected 1, 2, 4, and 24 h after the light exposure (453 nm). Bars represent the mean ± S.D. of 8 individual experiments (n = 8). *, *p* < 0.05 and compared to non-irradiated control cultures (white bars). (**C**) Exemplary microscopic photographs of neutral, red-treated control fibroblast cultures (control) as well as blue light-exposed cultures 1 and 24 h after light exposure with 80 J/cm^2^.

In addition to the ATP production rate data obtained through Seahorse^®^ technology, we characterized the native ATP concentration in irradiated and non-irradiated fibroblast cultures using an ATP detection kit. The patterns of ATP concentrations and the kinetics of ATP production after blue light exposure clearly correlated with the previously described effects of blue light exposure on cellular NAD^+^ and NADH concentration, as well as with the mitochondrial respiration and glycolysis data obtained through Seahorse^®^ technology (Figure 6A). Thus, we observed a clear and statistically significant decrease in cellular ATP production both in resting and TGF-β activated fibroblast cultures 1 and 4 h after light exposure. These reductions were, once again, reversible, and the ATP concentration returned to the levels of the untreated control at least 24 h after irradiation.

As an example of a potential biological consequence of cellular ATP depletion and to demonstrate the biological relevance of blue light-modified ATP metabolism, we evaluated the effect of blue light irradiation on a typical ATP-dependent cellular parameter, namely the endocytosis of the dye Neutral Red and lysosomal activity. In Figure 6A,C, we show that the endocytosis of Neutral Red and lysosomal activity are significantly reduced at least four hours after light exposure in a light dose-dependent manner (20–80 J/cm^2^). This process was reversible as well, and the light effect shows no significant changes after 24 h.

## 4. Discussion

Wound healing is a complex, dynamic, and interactive biological process that occurs in at least three overlapping phases. After an injury, various intra- and intercellular signaling pathways are activated and coordinated with different cell types to restore tissue integrity and homeostasis [39]. In the final, regenerative phase, the collagen matrix is continuously remodeled, the number of cells decreases, and the tensile strength of the scar is restored [40]. A key process in this phase is the differentiation of fibroblasts into myofibroblasts, induced by the TGF-β1 signaling pathway, which is crucial for wound closure through contractile forces [41,42,43]. Dysregulation of any of these phases can lead to complications in healing, potentially resulting in chronic wounds or excessive healing [44]. Excessive wound healing, caused by factors such as increased collagen synthesis or persistent mechanical stress, can manifest as hypertrophic scars and keloids [39,40,45]. These are classified as fibroproliferative diseases, often marked by a disrupted healing process [46,47]. They are characterized by excessive collagen deposition, sustained myofibroblast activity, and reduced apoptosis [2,48,49]. Myofibroblasts are the predominant cell type in fibrotic diseases [41,50].

Increased glycolysis significantly correlates with the manifestation of the fibrogenic phenotype in fibroblasts [51,52], explaining the higher proliferative capacity of fibroblasts in hypertrophic scars compared to normal skin fibroblasts [53]. Enhanced aerobic glycolysis also promotes myofibroblast activation, while direct or indirect inhibition of glycolysis significantly reduces TGF-β1-induced fibronectin and α-SMA protein expression and myofibroblast differentiation [23,30,52], positioning glycolysis inhibition as a potential anti-fibrotic therapy [23]. The enzyme 6-Phosphofructo-2-Kinase/Fructose-2,6-Bisphosphatase 3 (PFKFB3) plays a pivotal role in the metabolic reprogramming that drives fibroblast-to-myofibroblast differentiation, a hallmark of tissue fibrosis. PFKFB3 regulates the synthesis of fructose-2,6-bisphosphate, a potent allosteric activator of phosphofructokinase-1, a key rate-limiting enzyme in glycolysis. Through this mechanism, PFKFB3 enhances glycolytic flux and shifts cellular metabolism towards a highly glycolytic state. The landmark study by Xie et al. [26] demonstrated that PFKFB3 expression is significantly upregulated during fibroblast differentiation, particularly in response to TGF-β1, a classic profibrotic cytokine. This upregulation leads to increased glycolysis and intracellular accumulation of succinate, a metabolite known to stabilize hypoxia-inducible factor 1-alpha (HIF-1α). HIF-1α subsequently induces a wide range of profibrotic genes, reinforcing the myofibroblast phenotype. Importantly, pharmacological inhibition of PFKFB3 or genetic knockdown of the enzyme significantly reduced myofibroblast differentiation, as evidenced by decreased expression of α-SMA and collagen.

Recently, we substantiated the crucial role of glycolysis in fibroblast differentiation and extended this concept by showing the equally critical role of mitochondrial respiration. We demonstrated that inhibition of these metabolic pathways by CO_2_ resulted in a statistically significant and marked reduction in TGF-β-induced fibroblast differentiation [30]. Thus, inhibiting glucose metabolism in fibroblasts could potentially disrupt pro-fibrotic pathways [26]. This would lead to decreased ATP production, cell cycle arrest, impaired cell proliferation, and possibly apoptosis [28,29].

As shown here, the antifibrotic effect observed after exposure to non-toxic doses of blue light at 453 nm, first described by our group [8,33,34], appears to be based on a similar molecular mechanism. The reduction in TGF-β-induced α-SMA and EDA-FN expression after exposure to blue light correlates strongly and statistically significantly with a reversible inhibition of glycolysis, mitochondrial respiration, and, consequently, ATP production. However, we cannot conclusively determine which of the molecular processes triggered by blue light ultimately modulates the differentiation of fibroblasts into myofibroblasts. For a therapeutic effect to occur, photons must be absorbed by a chromophore within the tissue. For the wavelength of 453 nm used in our study, porphyrin-containing enzymes and flavoproteins serve as photo-acceptors [54,55]. Flavoproteins are enzymes that contain flavin molecules, such as flavin adenine dinucleotide (FAD) or flavin mononucleotide (FMN), yellow compounds with electrophilic and nucleophilic properties, functioning as coenzymes in numerous electron transfer reactions [55,56,57], many of which are involved in primary metabolic pathways, such as the citric acid cycle, β-oxidation, or the respiratory chain [58,59]. Their absorption ranges cover UVB, UVA, and blue light, with the maximum absorption of oxidized flavin adenine dinucleotide (FAD) in the blue spectrum at approximately 450 nm [55].

The NADH:ubiquinone oxidoreductase (Complex I) of the mitochondrial respiratory chain plays a central role in oxidative phosphorylation. It transfers electrons from NADH to ubiquinone (coenzyme Q) and couples this transfer to the translocation of protons across the inner mitochondrial membrane. In this process, FMN plays a key role as the primary electron acceptor. FMN is reduced by NADH to FMNH_2_, before the electrons are transferred via a series of iron–sulfur clusters (Fe–S centers) to ubiquinone [60]. This reaction releases free energy, which is used for the active transport of protons (H^+^) from the matrix into the intermembrane space, thereby establishing the electrochemical proton gradient, which in turn serves as the driving force for ATP synthesis via ATP synthase (Complex V) [61]. However, if FMN is artificially reduced, for example, by photochemical reduction and without the oxidation of NADH, the natural coupling of electron transfer and energy-dependent proton translocation is lost. In this case, the exergonic reaction of NADH oxidation, which normally drives the mechanical work of proton transport, is absent. Although the artificially reduced state of FMN could still transfer electrons to downstream components of the respiratory chain, this would occur uncoupled from proton pumping, resulting in a very weak proton gradient or none at all. This, in turn, inhibits ATP synthesis, as ATP synthase depends on this gradient [62]. Another important consequence concerns the cellular NAD^+^/NADH ratio. In the absence of NADH oxidation by complex I, NADH remains in its reduced form, and NAD^+^ is not regenerated. This leads to a reduction in the NAD^+^/NADH ratio, which profoundly affects numerous metabolic processes. Many dehydrogenases in the citric acid cycle and glycolysis are NAD^+^-dependent, including isocitrate dehydrogenase, α-ketoglutarate dehydrogenase, and glyceraldehyde-3-phosphate dehydrogenase (GAPDH). In conditions of NAD^+^ deficiency, these enzymatic reactions are inhibited, leading to a general slowdown of mitochondrial and cytosolic energy production [63]. Indeed, following light exposure at 453 nm, we observed a significant decrease in the NAD^+^/NADH ratio, primarily driven by a strong and statistically significant reduction in intracellular NAD^+^ levels. Thus, there is strong evidence suggesting that the reduction in glycolytic activity we observed may be a consequence of the photoreduction of mitochondrial flavoproteins. Since glycolysis depends on a steady supply of NAD^+^, especially for the glyceraldehyde-3-phosphate dehydrogenase (GAPDH) step, this leads to a metabolic bottleneck where glycolytic flux stalls despite upstream activation, such as through PFKFB3 [26,64].

Additionally, we noted the importance of ATP in impairing fibroblast-to-myofibroblast differentiation. We found that blue light-induced ATP deficiency strongly correlated with impaired lysosomal activity in the neutral red assay. The accumulation of neutral red in lysosomes is not directly ATP-dependent, as it does not occur via an active transport mechanism. However, the functionality of the lysosomal pH, and thus the ability of the dye to accumulate, is indirectly linked to the cellular ATP status. The maintenance of the acidic pH within the lysosomal lumen (~pH 4.5–5.0) is achieved through the activity of the V-type H^+^-ATPase, a proton-pumping ATPase that continuously transports protons into the lysosome using ATP [65]. Numerous processes within the endomembrane system, including endocytosis, the maturation of endosomes into lysosomes, and the fusion of lysosomal compartments, rely on ATP-dependent motor proteins and GTPases. Under energy-deprived conditions, there is a reduction in lysosome numbers, a slowing of vesicular transport, and disruptions in organelle morphology and function [66,67]. Lysosomes are no longer viewed solely as degradative organelles, but rather as central hubs for cellular signaling, metabolite sensing, and inter-organelle communication. Lysosomal acidification, enzyme trafficking, and autophagosome-lysosome fusion are all ATP-dependent steps. Without adequate ATP, lysosomal activity collapses, leading to impaired autophagic flux [68]. The ATP-dependent acidification of the lysosomal lumen is essential for the regulation of autophagy, mTOR signaling, and metabolic flexibility, all processes closely linked to fibroblast differentiation [69,70]. Autophagy, particularly via the MTORC2-CTGF axis, has been shown to be a prerequisite for fibroblast activation, enabling the clearance of damaged proteins and organelles, as well as the metabolic adaptation needed for myofibroblast transition. Disruption of autophagy blocks the expression of key differentiation markers such as α-SMA and collagen [68,71,72].

A sustained inhibition of energy metabolism would inevitably lead to cell death. However, under the irradiation conditions we used, we did not observe an increased rate of cell death in the irradiated fibroblast cultures. Generally, photochemical reduction is reversible, and reoxidation occurs via various chemical and biochemical mechanisms [73]. A central factor in this reoxidation is molecular oxygen (O_2_), which serves as an efficient electron acceptor for reduced flavins. In the presence of O_2_, so-called dark reoxidation frequently occurs, during which FMNH_2_ or FADH_2_ spontaneously donates electrons to O_2_. This reaction generates reactive oxygen species (ROS) such as superoxide (O_2_^•−^) or hydrogen peroxide (H_2_O_2_), while simultaneously restoring flavins to their oxidized state [74]. The kinetics of this process depend on variables such as oxygen partial pressure, flavin localization (soluble vs. membrane-bound), pH, and the presence of antioxidants. Additionally, cellular redox systems such as the thioredoxin or glutathione systems can modulate reoxidation. These systems either interact directly with flavin-containing enzymes or regulate the redox state of the cytosol and mitochondria, allowing for indirect control of flavin oxidation [75,76]. In the presence of NAD^+^-dependent dehydrogenases, enzymatic reoxidation of photoreduced flavins can also occur, particularly when flavins are tightly bound to proteins, as in NADPH oxidase or cytochrome P450 reductase [77,78]. Some studies have shown that photoreduced flavins may also exhibit complex reaction kinetics, for example, involving delayed or multi-phase reoxidation, suggesting the formation of intermediates or feedback-controlled effects. For instance, FMNH_2_ can react with nitric oxide (NO), oxygen, or lipid peroxides to form more stable but reversible complexes, which reoxidize only after a certain delay [79]. Our data show that the mitochondrial respiration impaired by light exposure, the significant reduction in glycolysis, the subsequent statistically significant decrease in ATP rate and NAD^+^/NADH ratio, as well as lysosomal activity, almost fully regenerate within less than 24 h after the last light exposure, without any signs of increased toxicity or other cell damage.

In conclusion, our findings demonstrate that blue light exposure at 453 nm induces a reversible suppression of key metabolic pathways, namely glycolysis, mitochondrial respiration, and ATP synthesis, most likely through the photoreduction of flavin-containing enzymes. This energy deficit disrupts NAD^+^ regeneration and lysosomal function, ultimately impairing the differentiation of fibroblasts into profibrotic myofibroblasts. Given the central role of myofibroblasts in pathological wound healing and fibrosis, these results highlight the potential of targeted photobiomodulation as a non-invasive, metabolism-based therapeutic strategy to prevent or control fibrotic skin disorders.

## Data Availability

The datasets used and/or analyzed during the current study are available from the corresponding author on reasonable request. Upon individual request, all data underlying this project can be made available to the interested parties.

## References

[1] Friedman S.L. (2004). Mechanisms of disease: Mechanisms of hepatic fibrosis and therapeutic implications. Nat. Clin. Pract. Gastroenterol. Hepatol..

[2] Tomasek J.J., Gabbiani G., Hinz B., Chaponnier C., Brown R.A. (2002). Myofibroblasts and mechano-regulation of connective tissue remodelling. Nat. Rev. Mol. Cell Biol..

[3] Quan T.E., Cowper S.E., Bucala R. (2006). The role of circulating fibrocytes in fibrosis. Curr. Rheumatol. Rep..

[4] Tomasek J.J., Schultz R.J., Episalla C.W., Newman S.A. (1986). The cytoskeleton and extracellular matrix of the Dupuytren’s disease “myofibroblast”: An immunofluorescence study of a nonmuscle cell type. J. Hand Surg. Am..

[5] Watanabe T., Barker T.A., Berk B.C. (2005). Angiotensin II and the endothelium: Diverse signals and effects. Hypertension.

[6] Brenner M., Herzinger T., Berking C., Plewig G., Degitz K. (2005). Phototherapy and photochemotherapy of sclerosing skin diseases. Photodermatol. Photoimmunol. Photomed..

[7] Kroft E.B.M., Berkhof N.J.G., van de Kerkhof P.C.M., Gerritsen M.M.J.P., de Jong E.M.G.J. (2008). Ultraviolet A phototherapy for sclerotic skin diseases: A systematic review. J. Am. Acad. Dermatol..

[8] Oplander C., Hidding S., Werners F.B., Born M., Pallua N., Suschek C.V. (2011). Effects of blue light irradiation on human dermal fibroblasts. J. Photochem. Photobiol. B.

[9] Wataya-Kaneda M., Ohno Y., Fujita Y., Yokozeki H., Niizeki H., Ogai M., Fukai K., Nagai H., Yoshida Y., Hamada I. (2018). Sirolimus Gel Treatment vs Placebo for Facial Angiofibromas in Patients with Tuberous Sclerosis Complex A Randomized Clinical Trial. JAMA Dermatol..

[10] Olson J.E., Stravino V.D. (1972). A review of cryotherapy. Phys. Ther..

[11] Schallhorn J.M., Schallhorn S.C., Hettinger K.A., Venter J.A., Pelouskova M., Teenan D., Hannan S.J. (2016). Outcomes and complications of excimer laser surgery in patients with collagen vascular and other immune-mediated inflammatory diseases. J. Cataract Refract. Surg..

[12] Kim S., Choi T.H., Liu W., Ogawa R., Suh J.S., Mustoe T.A. (2013). Update on scar management: Guidelines for treating Asian patients. Plast. Reconstr. Surg..

[13] El Ayadi A., Jay J.W., Prasai A. (2020). Current Approaches Targeting the Wound Healing Phases to Attenuate Fibrosis and Scarring. Int. J. Mol. Sci..

[14] Taheri A., Mansoori P., Al-Dabagh A., Feldman S.R. (2014). Are corticosteroids effective for prevention of scar formation after second-degree skin burn?. J. Dermatol. Treat..

[15] Nguyen J.K., Austin E., Huang A., Mamalis A., Jagdeo J. (2020). The IL-4/IL-13 axis in skin fibrosis and scarring: Mechanistic concepts and therapeutic targets. Arch. Dermatol. Res..

[16] Hosseini M., Brown J., Khosrotehrani K., Bayat A., Shafiee A. (2022). Skin biomechanics: A potential therapeutic intervention target to reduce scarring. Burns Trauma.

[17] Bernard K., Logsdon N.J., Ravi S., Xie N., Persons B.P., Rangarajan S., Zmijewski J.W., Mitra K., Liu G., Darley-Usmar V.M. (2015). Metabolic Reprogramming Is Required for Myofibroblast Contractility and Differentiation. J. Biol. Chem..

[18] Gibb A.A., Lazaropoulos M.P., Elrod J.W. (2020). Myofibroblasts and Fibrosis: Mitochondrial and Metabolic Control of Cellular Differentiation. Circ. Res..

[19] Vander Heiden M.G., Cantley L.C., Thompson C.B. (2009). Understanding the Warburg effect: The metabolic requirements of cell proliferation. Science.

[20] Bardon A., Ceder O., Kollberg H. (1986). Increased activity of four glycolytic enzymes in cultured fibroblasts from cystic fibrosis patients. Res. Commun. Chem. Pathol. Pharmacol..

[21] Henderson J., Duffy L., Stratton R., Ford D., O’Reilly S. (2020). Metabolic reprogramming of glycolysis and glutamine metabolism are key events in myofibroblast transition in systemic sclerosis pathogenesis. J. Cell. Mol. Med..

[22] Hewitson T.D., Smith E.R. (2021). A Metabolic Reprogramming of Glycolysis and Glutamine Metabolism Is a Requisite for Renal Fibrogenesis—Why and How?. Front. Physiol..

[23] Ding H., Jiang L., Xu J., Bai F., Zhou Y., Yuan Q., Luo J., Zen K., Yang J. (2017). Inhibiting aerobic glycolysis suppresses renal interstitial fibroblast activation and renal fibrosis. Am. J. Physiol. Ren. Physiol..

[24] Chen Y., Choi S.S., Michelotti G.A., Chan I.S., Swiderska-Syn M., Karaca G.F., Xie G., Moylan C.A., Garibaldi F., Premont R. (2012). Hedgehog controls hepatic stellate cell fate by regulating metabolism. Gastroenterology.

[25] Kottmann R.M., Kulkarni A.A., Smolnycki K.A., Lyda E., Dahanayake T., Salibi R., Honnons S., Jones C., Isern N.G., Hu J.Z. (2012). Lactic acid is elevated in idiopathic pulmonary fibrosis and induces myofibroblast differentiation via pH-dependent activation of transforming growth factor-beta. Am. J. Respir. Crit. Care Med..

[26] Xie N., Tan Z., Banerjee S., Cui H., Ge J., Liu R.M., Bernard K., Thannickal V.J., Liu G. (2015). Glycolytic Reprogramming in Myofibroblast Differentiation and Lung Fibrosis. Am. J. Respir. Crit. Care Med..

[27] Goodwin J., Choi H., Hsieh M.H., Neugent M.L., Ahn J.M., Hayenga H.N., Singh P.K., Shackelford D.B., Lee I.K., Shulaev V. (2018). Targeting Hypoxia-Inducible Factor-1alpha/Pyruvate Dehydrogenase Kinase 1 Axis by Dichloroacetate Suppresses Bleomycin-induced Pulmonary Fibrosis. Am. J. Respir. Cell Mol. Biol..

[28] Giammarioli A.M., Gambardella L., Barbati C., Pietraforte D., Tinari A., Alberton M., Gnessi L., Griffin R.J., Minetti M., Malorni W. (2012). Differential effects of the glycolysis inhibitor 2-deoxy-D-glucose on the activity of pro-apoptotic agents in metastatic melanoma cells, and induction of a cytoprotective autophagic response. Int. J. Cancer.

[29] Golding J.P., Wardhaugh T., Patrick L., Turner M., Phillips J.B., Bruce J.I., Kimani S.G. (2013). Targeting tumour energy metabolism potentiates the cytotoxicity of 5-aminolevulinic acid photodynamic therapy. Br. J. Cancer.

[30] Fleckner M., Dohmen N.K., Salz K., Christophers T., Windolf J., Suschek C.V., Oezel L. (2024). Exposure of Primary Human Skin Fibroblasts to Carbon Dioxide-Containing Solution Significantly Reduces TGF-beta-Induced Myofibroblast Differentiation In Vitro. Int. J. Mol. Sci..

[31] Hegmann L., Sturm S., Niegisch G., Windolf J., Suschek C.V. (2022). Enhancement of human bladder carcinoma cell chemosensitivity to Mitomycin C through quasi-monochromatic blue light (lambda = 453 ± 10 nm). J. Photochem. Photobiol. B.

[32] Sturm S., Niegisch G., Windolf J., Suschek C.V. (2024). Exposure of Bladder Cancer Cells to Blue Light (lambda = 453 nm) in the Presence of Riboflavin Synergistically Enhances the Cytotoxic Efficiency of Gemcitabine. Int. J. Mol. Sci..

[33] Krassovka J.M., Suschek C.V., Prost M., Grotheer V., Schiefer J.L., Demir E., Fuchs P.C., Windolf J., Sturmer E.K., Oplander C. (2020). The impact of non-toxic blue light (453 nm) on cellular antioxidative capacity, TGF-beta1 signaling, and myofibrogenesis of human skin fibroblasts. J. Photochem. Photobiol. B.

[34] Taflinski L., Demir E., Kauczok J., Fuchs P.C., Born M., Suschek C.V., Oplander C. (2014). Blue light inhibits transforming growth factor-beta1-induced myofibroblast differentiation of human dermal fibroblasts. Exp. Dermatol..

[35] Chavan H., Christudoss P., Mickey K., Tessman R., Ni H.M., Swerdlow R., Krishnamurthy P. (2017). Arsenite Effects on Mitochondrial Bioenergetics in Human and Mouse Primary Hepatocytes Follow a Nonlinear Dose Response. Oxid. Med. Cell. Longev..

[36] Butler M.G., Hossain W.A., Tessman R., Krishnamurthy P.C. (2018). Preliminary observations of mitochondrial dysfunction in Prader-Willi syndrome. Am. J. Med. Genet. A.

[37] Mookerjee S.A., Brand M.D. (2015). Measurement and Analysis of Extracellular Acid Production to Determine Glycolytic Rate. J. Vis. Exp..

[38] Kull F.C., Cuatrecasas P. (1983). Estimation of cell number by neutral red content. Applications for proliferative and survival assays. Appl. Biochem. Biotechnol..

[39] Gurtner G.C., Werner S., Barrandon Y., Longaker M.T. (2008). Wound repair and regeneration. Nature.

[40] Darby I.A., Laverdet B., Bonte F., Desmouliere A. (2014). Fibroblasts and myofibroblasts in wound healing. Clin. Cosmet. Investig. Dermatol..

[41] Barnes J.L., Gorin Y. (2011). Myofibroblast differentiation during fibrosis: Role of NAD(P)H oxidases. Kidney Int..

[42] Darby I., Skalli O., Gabbiani G. (1990). Alpha-smooth muscle actin is transiently expressed by myofibroblasts during experimental wound healing. Lab. Investig..

[43] Vaughan M.B., Howard E.W., Tomasek J.J. (2000). Transforming growth factor-beta1 promotes the morphological and functional differentiation of the myofibroblast. Exp. Cell Res..

[44] Broughton G., Janis J.E., Attinger C.E. (2006). Wound healing: An overview. Plast. Reconstr. Surg..

[45] Broughton G., Janis J.E., Attinger C.E. (2006). The basic science of wound healing. Plast. Reconstr. Surg..

[46] Lee H.J., Jang Y.J. (2018). Recent Understandings of Biology, Prophylaxis and Treatment Strategies for Hypertrophic Scars and Keloids. Int. J. Mol. Sci..

[47] Wolfram D., Tzankov A., Pulzl P., Piza-Katzer H. (2009). Hypertrophic scars and keloids—A review of their pathophysiology, risk factors, and therapeutic management. Dermatol. Surg..

[48] Reinke J.M., Sorg H. (2012). Wound repair and regeneration. Eur. Surg. Res..

[49] Singer A.J., Clark R.A. (1999). Cutaneous wound healing. N. Engl. J. Med..

[50] Pakshir P., Noskovicova N., Lodyga M., Son D.O., Schuster R., Goodwin A., Karvonen H., Hinz B. (2020). The myofibroblast at a glance. J. Cell Sci..

[51] Chen Z., Wang Z., Jin T., Shen G., Wang Y., Tan X., Gan Y., Yang F., Liu Y., Huang C. (2019). Fibrogenic fibroblast-selective near-infrared phototherapy to control scarring. Theranostics.

[52] Wei Q., Su J., Dong G., Zhang M., Huo Y., Dong Z. (2019). Glycolysis inhibitors suppress renal interstitial fibrosis via divergent effects on fibroblasts and tubular cells. Am. J. Physiol. Ren. Physiol..

[53] Ghazawi F.M., Zargham R., Gilardino M.S., Sasseville D., Jafarian F. (2018). Insights into the Pathophysiology of Hypertrophic Scars and Keloids: How Do They Differ?. Adv. Skin Wound Care.

[54] Losi A. (2007). Flavin-based Blue-Light photosensors: A photobiophysics update. Photochem. Photobiol..

[55] Losi A., Gartner W. (2011). Old chromophores, new photoactivation paradigms, trendy applications: Flavins in blue light-sensing photoreceptors. Photochem. Photobiol..

[56] Massey V. (2000). The chemical and biological versatility of riboflavin. Biochem. Soc. Trans..

[57] Iwata T., Masuda S. (2021). Photoreaction Mechanisms of Flavoprotein Photoreceptors and Their Applications. Adv. Exp. Med. Biol..

[58] Ahmad M., Wolberg A., Kahwaji C.I. (2025). Biochemistry, Electron Transport Chain. StatPearls.

[59] Serrage H., Heiskanen V., Palin W.M., Cooper P.R., Milward M.R., Hadis M., Hamblin M.R. (2019). Under the spotlight: Mechanisms of photobiomodulation concentrating on blue and green light. Photochem. Photobiol. Sci..

[60] Hirst J. (2013). Mitochondrial complex I. Annu. Rev. Biochem..

[61] Rich P.R., Marechal A. (2010). The mitochondrial respiratory chain. Essays Biochem..

[62] Wikstrom M.K. (1977). Proton pump coupled to cytochrome c oxidase in mitochondria. Nature.

[63] Ying W. (2008). NAD^+^/NADH and NADP^+^/NADPH in cellular functions and cell death: Regulation and biological consequences. Antioxid. Redox Signal..

[64] Yan S., Wei X., Xu S., Sun H., Wang W., Liu L., Jiang X., Zhang Y., Che Y. (2017). 6-Phosphofructo-2-kinase/fructose-2,6-bisphosphatase isoform 3 spatially mediates autophagy through the AMPK signaling pathway. Oncotarget.

[65] Mindell J.A. (2012). Lysosomal acidification mechanisms. Annu. Rev. Physiol..

[66] Luzio J.P., Pryor P.R., Bright N.A. (2007). Lysosomes: Fusion and function. Nat. Rev. Mol. Cell Biol..

[67] Saftig P., Klumperman J. (2009). Lysosome biogenesis and lysosomal membrane proteins: Trafficking meets function. Nat. Rev. Mol. Cell Biol..

[68] Bernard M., Dieude M., Yang B., Hamelin K., Underwood K., Hebert M.J. (2014). Autophagy fosters myofibroblast differentiation through MTORC2 activation and downstream upregulation of CTGF. Autophagy.

[69] Settembre C., Fraldi A., Medina D.L., Ballabio A. (2013). Signals from the lysosome: A control centre for cellular clearance and energy metabolism. Nat. Rev. Mol. Cell Biol..

[70] Perera R.M., Zoncu R. (2016). The Lysosome as a Regulatory Hub. Annu. Rev. Cell Dev. Biol..

[71] Saxton R.A., Sabatini D.M. (2017). mTOR Signaling in Growth, Metabolism, and Disease. Cell.

[72] Araya J., Kojima J., Takasaka N., Ito S., Fujii S., Hara H., Yanagisawa H., Kobayashi K., Tsurushige C., Kawaishi M. (2013). Insufficient autophagy in idiopathic pulmonary fibrosis. Am. J. Physiol. Lung Cell. Mol. Physiol..

[73] Massey V., Hemmerich P. (1978). Photoreduction of flavoproteins and other biological compounds catalyzed by deazaflavins. Biochemistry.

[74] Kussmaul L., Hirst J. (2006). The mechanism of superoxide production by NADH:ubiquinone oxidoreductase (complex I) from bovine heart mitochondria. Proc. Natl. Acad. Sci. USA.

[75] Holmgren A. (1989). Thioredoxin and glutaredoxin systems. J. Biol. Chem..

[76] Schafer F.Q., Buettner G.R. (2001). Redox environment of the cell as viewed through the redox state of the glutathione disulfide/glutathione couple. Free Radic. Biol. Med..

[77] Hamdane D., Xia C., Im S.C., Zhang H., Kim J.J., Waskell L. (2009). Structure and function of an NADPH-cytochrome P450 oxidoreductase in an open conformation capable of reducing cytochrome P450. J. Biol. Chem..

[78] Pandey A.V., Fluck C.E. (2013). NADPH P450 oxidoreductase: Structure, function, and pathology of diseases. Pharmacol. Ther..

[79] Bruice T.C., Benkovic S.J. (2000). Chemical basis for enzyme catalysis. Biochemistry.

